# Temporal and Spatial Population Genetic Variation in Chilean Jack Mackerel (*Trachurus murphyi*)

**DOI:** 10.3390/biology14050510

**Published:** 2025-05-07

**Authors:** Cristian B. Canales-Aguirre, Sandra Ferrada Fuentes, Ricardo Galleguillos

**Affiliations:** 1 Centro i~mar, Universidad de Los Lagos, Camino a Chinquihue 6 km, Puerto Montt 5480000, Chile; 2 Laboratorio de Genética y Acuicultura, Departamento de Oceanografía, Facultad de Ciencias Naturales y Oceanográficas, Universidad de Concepción, Concepción 4070386, Chile; sferrada@udec.cl (S.F.F.); rgallegui@gmail.com (R.G.); 3 Doctorado en Sistemática y Biodiversidad, Facultad de Ciencias Naturales y Oceanográficas, Universidad de Concepción, Concepción 4070386, Chile

**Keywords:** Chilean jack mackerel, genetic homogeneity, jurel, microsatellites, temporal genetic variation

## Abstract

*Trachurus murphyi* have been studied for population genetic structures for decades, identifying only one large population across the South Pacific Ocean. Although all of these studies have extensively examined the spatial genetic pattern, there remains a gap in understanding the potential role of temporality. Our study aims to elucidate spatial and temporal genetic patterns in *T. murphyi* populations in the South Pacific Ocean, examining genetic composition across seasons, including feeding and spawning seasons, where the latter was not previously investigated. Using 10 microsatellite loci, our study confirms an overall consistent and stable population genetic pattern in *T. murphyi* across its geographic distribution observed over multiple years and seasons. The only exception was found for New Zealand in the spring–summer season. Furthermore, we identify potential genetic markers for monitoring variability in the species.

## Simple Summary

Scientists have studied the population genetics of *Trachurus murphyi* (Chilean jack mackerel) for decades and found that it forms a single large population across the South Pacific Ocean. While previous research has focused on genetic differences across locations, little is known about how these patterns change over time. Our study explores both regional and seasonal genetic patterns in *T. murphyi*, including differences between feeding and spawning seasons—something not previously examined. By analyzing genetic markers, we confirm that the species maintains an overall stable genetic structure across different years and seasons. Additionally, we identify potential genetic markers that could help monitor future changes in the population.

## 1. Introduction

The Chilean jack mackerel, *Trachurus murphyi*, is a highly mobile marine fish distributed throughout the South Pacific Ocean (SPO) [1]. It is found along the coast from Ecuador to Chile in the SPO and near the coasts of New Zealand and the Tasmanian Sea [1,2,3]. Due to its significant importance for fisheries in the SPO region, numerous studies have investigated population differences within its distribution range, examining various aspects such as life history traits, meristic and morphometric characteristics, and microchemistry of otoliths, as well as genetic markers [1,3,4,5,6,7,8,9,10,11,12,13].

Several studies have been conducted using molecular tools to obtain insight into the population genetic structure in *T. murphyi* across the SPO. Since the 1980s, all of them have focused on the integration of population genetic data into a tool for informed decision-making for this species [4,5,7]. For instance, Cárdenas et al. [5] found high genetic diversity using three heterologous microsatellites, and similar results were found by Ferrada Fuentes et al. [7] using seven microsatellites. These population genetic studies and others have concluded that this species has not shown population genetic structure along its geographic distribution. However, while these studies have extensively examined spatial genetic patterns, there remains a gap in understanding the potential role of temporality. It is important to consider that individuals may migrate to spawning areas before dispersing to feeding areas, where mixing occurs [14,15]. Therefore, sampling individuals in spawning areas can provide valuable insights and maximize the likely population genetic difference compared with areas where there can be mixing.

Our study aims to shed new light on the spatial and temporal genetic composition of *T. murphyi* populations in the SPO. We study genetic patterns over different seasons across a year, including feeding (winter–fall) and spawning (spring–summer), which were not included in previous studies. As a null hypothesis (H0), we propose that there are no significant genetic differences over time (between seasons) or across space (between feeding and spawning areas). Alternatively (H1), we hypothesize that genetic differences will be more pronounced during the spawning season (spring–summer) due to population segregation in spawning areas, whereas no genetic structuring is expected during the feeding season (fall–winter) due to population mixing. Additionally, we expect to identify genetic markers that may serve as indicators of changes in genetic diversity by comparing the expected heterozygosity with previous studies that used the same loci. If the population structure remains stable, heterozygosity levels should be similar; otherwise, deviations could suggest recent evolutionary processes such as genetic drift or shifts in migration patterns. Finally, we aim to expand our understanding of population structures in marine species over time and shed light on the evolutionary processes within *T. murphyi*.

## 2. Materials and Methods

### 2.1. Sample Collection Areas

**Figure 1:**
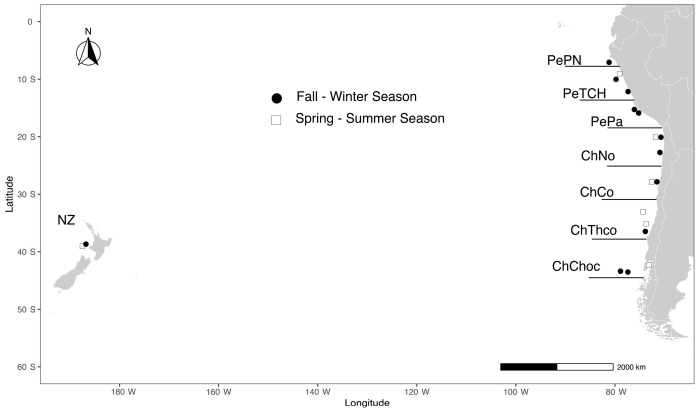
Sampling locations within areas in fall–winter (black dots) and spring–summer (white squares) for *Trachurus murphyi*. Locations from Peru have names starting with “Pe” while those from Chile start with “Ch”.

We collected a total of 852 individuals of *Trachurus murphyi* in 2011–2012 from 18 locations across the coastal areas of the SPO (i.e., Peru, Chile, and New Zealand; Figure 1) during fall–winter (_FW, N = 538) and spring–summer (_SS, N = 314) seasons. The fall–winter season (March to August) corresponds to the main period where fishery vessels catch Chilean jack mackerel; hence, there are no biological reproductive closures. Also, this period corresponds to the feeding time, so individuals disperse from their main reproductive areas to several points along the coast and ocean zones. The fall–winter season included twelve locations: six from Peru, five from Chile, and one from New Zealand. The fall–winter locations were aggregated as follows: PePN_FW (Pimentel), PeTCH_FW (Chimbote and Ancon), and PePa_FW (Cañete, Tambo de Moras, and Olleros) in Peru; ChNo_FW (Iquique and Mejillones), ChCo_FW (Coquimbo), ChThco_FW (Talcahuano coast), and ChChoc_FW (Chiloé oceanic) in Chile; and NZ_FW in New Zealand. The spring–summer season (October to February) corresponds to the spawning season, where fishery vessels are forbidden to catch Chilean jack mackerel [1] because of biological reproductive closures. This season, individuals move from feeding to their main reproductive areas [1]. The spring–summer season included six locations: one from Peru, four from Chile, and one from New Zealand. The spring–summer locations were aggregated as follows: PeTCH_SS (Chiclayo) in Peru; ChNo_SS (Iquique), ChCo_SS (Coquimbo), ChThco_SS (Talcahuano coast), and ChChoc_SS (Puerto Montt) in Chile; and NZ_SS in New Zealand. All previous genetic studies conducted on *T. murphyi* have collected samples from the fall–winter season because accessibility is easier. Here, we included the spawning season of Chilean jack mackerel samples to enhance the robustness in delineating population genetic structure [14], as well as the feeding season, anticipating the mixing of individuals from different areas.

### 2.2. Laboratory Procedures

For all sampled individuals, we obtained a piece of muscular tissue that was preserved in 96% ethanol for subsequent analyses. The extraction and isolation of the total genomic DNA were carried out using a salting-out protocol as described by Jowett [16]. We assessed the quality and quantity of each DNA purification with an Eppendorf biophotometer^®^ (Hamburg, Germany), diluting the template DNA to 20 ng/µL for further PCR amplification. We amplified ten microsatellite loci where three correspond to heterologous loci from *Trachurus trachurus* (Tt29, Tt62, and Tt133, [17]), and seven correspond to species-specific loci from *T. murphyi* (Tmur A101, Tmur A104, Tmur A115, Tmur B2, Tmur B6, TmurB104, and Tmur C4 [4]). The concentrations of reagents for PCR for the heterologous loci were as follows: 1.5 mM of MgCl_2_, 0.2 mM of dNTPs, 0.2 µM of each primer (the 3′ end of the forward primer included a dye), and 0.1 U/µL of Taq DNA polymerase (Invitrogen^®^, CAR, Burlington, ON, Canada). The thermocycler conditions were similar to those described in Kasapidis and Magoulas [17], except that we standardized the annealing temperature to 58 °C for all loci. For the species-specific loci, the concentrations of reagents for PCR and thermocycler conditions were conducted following Canales-Aguirre et al. [4]. All PCRs were conducted using a PTC-200 (MJ-Research^®^, Saint-Bruno-de-Montarville, QC, Canada) thermocycler, and we included both positive and negative controls. Amplicons were visualized with an ethidium-bromide-stained gel. A fragment analysis was conducted for PCR products on an ABI 3330 DNA sequencer, and we scored alleles using Peak ScannerTM software v1.0, with GS500 as the internal size standard.

### 2.3. Analysis Procedures

For summary statistics of genetic diversity, we calculated the total number of alleles (N_A_) and the expected (H_E_) and observed (H_O_) heterozygosity for each locus and area using GENALEX v6.5 software [18].

For testing spatial and temporal structure, we conducted a Principal Component Analysis (PCA), calculated the pairwise F_ST_ indices, and performed an analysis of molecular variance (AMOVA). PCA was used to reduce the multivariate microsatellite multilocus data and provide insights into the genetic diversity among the sampled individuals. All samples were included to visualize both the geographical and temporal patterns underlying seasonal variation. Pairwise F_ST_ indices were calculated following Weir and Cockerham [19] using the Stampp v1.3.6 R package [20], including all areas and seasons. The results were then visualized in a heatmap using the ggplot2 v3.5.1 R package [21]. The AMOVA was performed to test different hypotheses (spatial groupings) of population differentiation that were obtained through non-genetic approaches. The hypotheses tested were (a) panmixia (genetic homogeneity between areas); (b) two separate populations in the SPO as suggested by non-genetic approaches, e.g., [1,8,9]; and (c) three separate populations considering two in the Southeastern Pacific Ocean and one in the Southwestern Pacific Ocean (i.e., New Zealand). We also conducted an AMOVA to estimate if there is a temporal structure among seasons. For both the pairwise F_ST_ comparison and AMOVA, *p*-values were obtained after 10,000 bootstraps, and sequential Bonferroni correction [22] for multiple comparisons was applied when necessary.

Finally, we used all previously published genetic information to compare the expected heterozygosity estimated in this study and previous research, considering the sampling collection year and loci tested. We used results from Cárdenas et al. [5] and Ferrada Fuentes et al. [7], which used similar geographical areas and loci. The expected heterozygosity was used because it is less sensitive to sample size imbalance than observed heterozygosity [23].

## 3. Results

*Analysis Procedures*

The summary statistics varied depending on the season and area (Table 1). For the fall–winter season, N_A_ ranged from 8.2 to 201, while for the spring–summer season, it ranged from 11.3 to 16.9 alleles by locus. Overall, N_A_ in the spring–summer was larger than in the fall–winter season. The observed heterozygosity for the fall–winter season ranged from 0.572 to 0.764, while for the spring–summer season, it ranged from 0.529 to 0.74. The expected heterozygosity for the fall–winter season ranged from 0.701 to 0.839, while for the spring–summer season, it ranged from 0.633 to 0.832.

**Figure 2:**
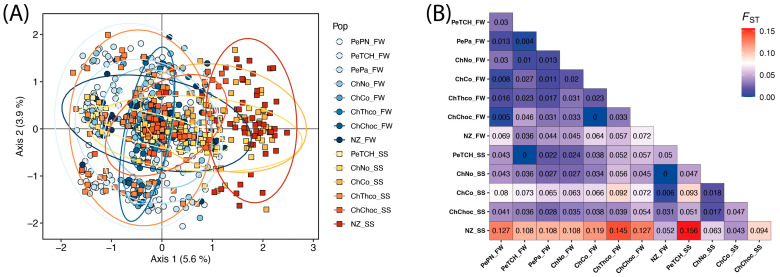
Genetic structure of sampled populations for *Trachurus murphyi*. (**A**) Principal Component Analysis (PCA) of multilocus microsatellite data showing genetic variation among individuals across different areas and seasons. Each point represents an individual, colored according to its geographic area, with different shapes indicating seasonal sampling: cold-colored circles for fall–winter (FW) and warm-colored squares for spring–summer (SS). Ellipses represent the 95% confidence interval for each area, highlighting the dispersion and potential genetic clustering of groups. The PCA axes indicate the percentage of total genetic variation explained. (**B**) Pairwise F_ST_ matrix quantifying genetic differentiation among sampled areas. The heatmap, generated using the ggplot2 R package, represents F_ST_ values, where darker shades of red indicate higher genetic differentiation, and blue shades indicate lower differentiation.

Overall, the PCA showed overlapping individuals from both geographical and temporal data, except for NZ_SS, where their dispersion is somewhat separated from the rest, but not for all (Figure 2A). The proportion of the variance explained by both axes was 9.5% (Axis 1 equal to 5.6 and Axis 2 equal to 3.9). Pairwise F_ST_ values were generally low (Figure 2B), with an overall average of 0.054. Within seasons, fall–winter showed an average F_ST_ of 0.029, while spring–summer showed a higher average of 0.077. The NZ_SS area showed pairwise F_ST_ values greater than 0.1 (ranging from 0.108 to 0.145) when compared to all fall–winter areas, except for NZ_FW (F_ST_ = 0.052). Compared to spring–summer areas, NZ_SS exhibited a particularly high value with PeCH_SS (F_ST_ = 0.156), the northernmost area.

The hierarchical analysis of molecular variance for several hypotheses did not show significant differences within each season (Table 2), and the F_ST_ and some F_CT_ in the AMOVA were negative. When testing non-differentiation between the fall–winter and spring–summer seasons in the AMOVA, we obtained an F_ST_ = −0.01855 (*p*-value = 1).

The comparisons of the expected heterozygosity among studies showed qualitative differences according to the season in this study, the year of the sampling location, and the locus tested (Table 3). Among studies, loci that did not vary considerably (<0.1 magnitude) were TmurA104, TmurA115, TmurC4, and Tt62. The loci TmurB6, TmurA101, Tt29, and Tt133, which showed a qualitative decline in the H_E_ during the spring–summer season from 2007 to 2012. Conversely, the H_E_ qualitative decreases in the fall–winter season were for TmurB104 and TmurB2.

## 4. Discussion

Research on *Trachurus murphyi* populations in the South Pacific Ocean has mostly focused on fishery data from the non-reproductive season. Previous studies have revealed genetic homogeneity across this region, which agrees with our findings. Interestingly, we also noticed a consistent pattern in the fall–winter and spring–summer seasons. This could indicate a regular movement of individuals between feeding and spawning areas along the coast of the SPO, potentially leading to genetic structuring. More research is needed, especially regarding microsatellite loci, to fully understand this phenomenon. From 2007 to 2012, a noteworthy aspect was the identification of four loci with diminished levels of expected heterozygosity during the spring–summer season and two during the fall–winter season. However, due to the limited number of overlapping genetic markers across studies, these findings should be interpreted with caution. While these specific genetic markers may serve as effective indicators for monitoring changes in genetic diversity, the limited overlap in loci makes comparative analyses of genetic diversity indices challenging, thus limiting comprehensive historical insights into genetic changes over time. Finally, this research calls into question the idea that differences in time, such as spawning and feeding seasons, may result in distinct temporal population structures.

Our study has shown that there is an overall lack of spatial population structure both within each season (fall–winter and spring–summer) and between them (with the only exception being NZ_SS), with low pairwise F_ST_ values and AMOVA results (*p* < 0.05). This supports previous research that found that marine fish populations often have F_ST_ values below 0.01 [24]. These results are also consistent with studies conducted on *T. murphyi* in the same region, using different markers such as isozymes and RFLP-PCR [25], mitochondrial DNA [5], and heterologous and species-specific microsatellites [4,5,7]. In the SPO, these findings are not uncommon and have been previously observed in other species within the same region. Additionally, a similar trend of genetic similarity was noticed in other marine species distributed across this area, including *Sprattus fuegensis*, *Genypterus blacodes*, and *Dissostichus eleginoides* [26,27,28,29]. These findings suggest that geographical barriers may not significantly drive divergence within this particular region.

Marine fish species, such as *T. murphyi*, typically possess several characteristics that contribute to maintaining genetic similarity among populations. These include large numbers of individuals, a tendency to reproduce and develop in the open ocean, high reproductive rates, and the ability to migrate. These factors have been extensively studied and have been found to play a crucial role in minimizing genetic divergence among populations [1,30]. The population dynamics of *T. murphyi* are heavily influenced by factors such as population size and the formation of reproductive aggregations, which occur in the SPO, where the species is ubiquitously found. These aggregations are known to occur throughout the species’ entire range [31], indicating that the species is highly abundant and displays extensive migratory behaviors. Additionally, current research has revealed that *T. murphyi* prefers to inhabit areas with specific temperature ranges between 15 and 18 °C [6]. Different intensities of El Niño events strongly impact the habitat patterns of commercially valuable species off the Chilean coast, altering suitable areas and distribution [32]. This could potentially lead to genetic mixing across the entire geographic range of the species. Overall, our research shows no genetic differentiation in *T. murphyi* over space and time throughout its distribution. Despite considerable distances between sample locations, the ability of a small number of individuals to migrate and successfully reproduce is enough to maintain genetic homogeneity within the population. Furthermore, similar results were found in *T. murphyi* relatives, *T. japonicus* [33] and *T. trecae* [34]. In *T. japonicus*, SNP markers showed no genetic differentiation around the Japanese archipelago [33], while *T. trecae*, using microsatellites, also lacked differentiation between temporal sample collections [34].

Upon analysis, the ten loci examined displayed polymorphism throughout their geographical range. When comparing the mean summary statistics of genetic diversity to those reported by DeWoody and Avise [35] for marine fish, we noted that the number of alleles for fall–winter (N_A_ = 13.8) and spring–summer (N_A_ = 14.9) samples fell below the average (N_A_ = 20.4). Similarly, the observed averages for heterozygosity in fall–winter (0.672) and spring–summer (0.646) were lower than the averages reported for other marine species (H_O_ = 0.790), indicating lower levels of genetic diversity in our study. The results of our study on *T. murphyi* have shown varying outcomes compared to previous research. While our estimation of expected heterozygosity (H_E_) using the same heterologous loci was generally low in the spring–summer season (two out of three loci) and showed no distinct pattern in the fall–winter season (one out of three loci), it is important to acknowledge the limited number of shared heterologous loci available for comparison [5]. Furthermore, upon examining the findings of Ferrada Fuentes et al. [7], we discovered that over 60% of the loci displayed low H_E_ in both seasons, with five out of six comparisons exhibiting lower values in the spring–summer season and four out of six in the fall–winter season. This highlights the need for caution in drawing definitive conclusions, given the scarcity of shared heterologous or specific loci for accurate comparison.

Our research uncovered fluctuations in expected heterozygosity at specific loci over time. Notably, expected heterozygosity, determined by allele frequencies, is more reliable than observed heterozygosity regarding sample size bias. This is because observed heterozygosity relies on the genotypes of individual samples [23]. Previous studies on the population genetics of *T. murphyi* have mostly relied on data collected during a single season. This has left gaps in our understanding of temporal population trends, especially during reproductive and non-reproductive phases. The motivation for this method stems from the idea that migration to certain reproductive areas could result in greater variety and changes in genetic diversity between seasons. Waples [15] proposes that sampling during the reproductive season is ideal for understanding population genetic patterns and reducing bias from related individuals [15,36]. While a meta-analysis of previous databases was not conducted, examining the expected heterozygosity over time offered valuable insight. Focusing on the fall–winter and spring–summer seasons provided a unique outlook on temporal diversity within a given year. Although variations in expected heterozygosity in certain loci from 2007 to 2012 indicated their potential for monitoring genetic diversity, overall differences were not significant enough to suggest a temporal population structure. However, some loci, such as TmurB6, did exhibit qualitative variation across both seasons.

Although this study’s results are consistent with prior research, it is crucial to consider certain limitations. The exclusive use of samples from active fishery vessels introduces potential variations in sampling locations, resulting in the need for wider geographical ranges rather than precise sites. As a result, certain regions in Peru were inaccessible for sampling, which could affect comparisons. Additionally, previous studies on *T. murphyi* have used partially overlapping loci in their microsatellite analyses, making direct comparisons of expected heterozygosity challenging.

Despite these limitations, this study incorporated data from three distinct years (2007–2012), supporting the temporal reliability of the results. However, we acknowledge that the temporal sampling is limited to a short period (2011–2012; fall–winter and spring–summer seasons), which may not capture slower genetic processes or important interannual changes for population trends. We believe a broader temporal scope is necessary to understand long-term genetic changes. Therefore, we suggest future studies include long-term monitoring to validate the genetic homogeneity detected in our study and further explore genetic changes over time.

The F_ST_ values raise concerns due to unequal sample sizes, highlighting potential issues with sample representativeness. Our data sets ranged from 38 to 135 for the fall–winter samples and from 48 to 72 for the spring–summer samples. We recognize that this bias could affect the robustness of genetic structure estimates (F_ST_ and AMOVA), but the bootstrapping procedure (i.e., 10,000 replicates) provides confidence in the results shown here. Some F_ST_ values were negative, which is common and reported as F_ST_ = 0, indicating greater genetic variation within populations than between them [19]. Negative F_ST_ values are statistically expected under panmixia, as they reflect stochastic noise when within-population diversity exceeds total diversity [19,37,38]. In our case, this supports the conclusion of genetic homogeneity in *T. murphyi* across areas, seasons, and years, suggesting a stable, undifferentiated population. AMOVA-based F_CT_/F_ST_ estimates can fluctuate due to limited sampling (e.g., few loci or individuals), but permutation tests in ARLEQUIN confirmed non-significance (*p* > 0.05), reinforcing that these values represent biological homogeneity rather than technical artifacts. Similar patterns are common in species with high gene flow or recent colonization histories [39]. Therefore, further sampling efforts may be necessary to ensure balanced sample sizes. However, the consistency of negative/zero F_ST_ values across temporal and spatial comparisons strongly supports our hypothesis of a panmictic population. Finally, we acknowledge that our conclusions heavily rely on the quantity of data and methods used. Although more sophisticated analysis might reveal some additional genetically organized data, we believe any recognized pattern would be minimal compared to the main conclusions we have reached.

To gain a comprehensive understanding of genetic differences, future analyses must prioritize the utilization of single-nucleotide polymorphisms (SNPs) obtained through reduced-representation sequencing techniques [40], such as RADseq, ddRAD, or DARTseq [41,42,43]. These methods have demonstrated their effectiveness in distinguishing genetic variations, particularly in species with relatively shallow genetic structures and high rates of gene flow, even on a small geographical scale [44]. These advanced techniques allow researchers to explore hypotheses surrounding neutral and adaptive genetic variation. For instance, studying neutral loci may uncover patterns of isolation by distance, e.g., [45], a particularly relevant consideration given the expansive range of *T. murphyi* throughout the Southeastern Pacific Ocean and Southwestern Pacific Ocean (~10,000 km). Adaptive loci could offer valuable insights into genetic differentiation linked to local adaptation in the species’ most extreme distribution areas. In addition, analyzing the entire genome over multiple years of data collection could further reinforce and validate the conclusions drawn from this study.

## 5. Conclusions

Our research supports the concept that *T. murphyi* maintains a stable, homogeneous genetic structure across all its inhabited areas, with no temporal differentiation observed. By analyzing specimens collected during both mating and non-mating seasons over multiple years in consistent settings, we have addressed a crucial gap in the current literature regarding temporal genetic variation. Additionally, the loci with reduced heterozygosity identified in this study could serve as valuable indicators for monitoring changes in genetic variability, particularly under scenarios of climate change or fishing pressure.

## Figures and Tables

**Table 1:** Mean summary statistics of genetic diversity by area for fall–winter and spring–summer seasons inferred for *Trachurus murphyi*.

	Fall–Winter Season				Spring–Summer Season			
Area	N	N_A_	H_O_	H_E_	N	N_A_	H_O_	H_E_
PePN	40	12.1	0.68	0.751	-	-	-	-
PeTCH	101	16.6	0.697	0.824	48	11.3	0.529	0.633
PePa	135	20.1	0.764	0.839	-	-	-	-
ChNo	96	15.7	0.73	0.825	48	16.3	0.74	0.818
ChCo	38	11.6	0.615	0.775	48	15.2	0.658	0.77
ChThco	40	8.2	0.572	0.701	72	15.3	0.637	0.739
ChChoc	40	13.6	0.703	0.764	48	16.9	0.723	0.832
NZ	48	12.4	0.612	0.728	50	14.6	0.591	0.689
Average		13.8	0.672	0.776		14.9	0.646	0.747

N, number of individuals; N_A_, average of the number of alleles per locus; H_O_, average of the observed heterozygosity; H_E_, average of the expected heterozygosity; -, no data for that area.

**Table 2:** Hierarchical analysis of molecular variance (AMOVA) to test for spatial variation by areas ^1^ in fall–winter and spring–summer inferred for *Trachurus murphyi*.

		Fall–Winter Season				Spring–Summer Season			
Nº Groups	Aggrupation	F_ST_	*p*-Value	F_CT_	*p*-Value	F_ST_	*p*-Value	F_CT_	*p*-Value
Three	Peru v/s Chile v/s NZ	−0.103	1	0	0.689	−0.007	1	0.05	0.133
Two	Peru + ChNo v/s Chile	−0.14	1	−0.074	0.973	−0.031	1	−0.005	0.604
One	Panmixis	−0.103	1	-	-	−0.030	1	-	-

^1^ Peru includes PePN, PeTCH, and PePa; Chile includes ChNo, ChCo, ChThco, and ChChoc. NZ is New Zealand. Peru + ChNo includes all areas in Peru and only the north of Chile (i.e., ChNo).

**Table 3:** Expected heterozygosity comparisons for this study and previous research, considering the sampling collection year and loci tested.

		Expected Heterozygosity									
Season/Previous Studies	Year	TmurB6	TmurA104	TmurA115	TmurC4	TmurA101	TmurB104	TmurB2	Tt29	Tt62	Tt133
Ferrada Fuentes et al. [7]	2007	-	0.943	0.95	-	0.927	-	-	0.747	0.855	0.861
Cárdenas et al. [5]	2009	-	-	-	-	-	-	-	0.76	0.83	0.87
Fall–Winter	2011	0.782	0.93	0.916	0.82	0.871	0.521	0.552	0.758	0.851	0.758
Spring–Summer	2011–2012	0.444	0.942	0.945	0.819	0.779	0.648	0.704	0.634	0.865	0.687

## Data Availability

The data presented in this study are available at https://github.com/Canales-AguirreCB/Canales-Aguirre_etal_2025_Biology (accessed on 10 March 2025).

## Abbreviations

The following abbreviations are used in this manuscript:

SPO	South Pacific Ocean
PCR	Polymerase Chain Reaction
DNA	Deoxyribonucleic Acid
AMOVA	Analysis of Molecular Variance
